# Isoflavone Attenuates the Caspase-1 and Caspase-3 Level in Cell Model of Parkinsonism

**DOI:** 10.1155/2015/725897

**Published:** 2015-06-16

**Authors:** Jian-xin Xu, Hai-ping Song, Qing-Xia Bu, De-Peng Feng, Xiao-Fan Xu, Qian-Ru Sun, Xue-Li Li

**Affiliations:** ^1^Department of Neurology, Liaocheng People's Hospital and Liaocheng Clinical School of Taishan Medical University, Liaocheng, Shandong 252000, China; ^2^Department of General Surgery, The Affiliated Union Hospital of Tongji Medical College, Huazhong University of Science and Technology, Wuhan, Hubei 430022, China; ^3^Department of Neuroimmune Laboratory, Liaocheng People's Hospital and Liaocheng Clinical School of Taishan Medical University, Liaocheng, Shandong 252000, China

## Abstract

The study has investigated the effect of isoflavone attenuates the caspase-1 and caspase-3 level in cell model of Parkinsonism. The subjects were PC12 cells. They were randomly divided into six groups: control, MPP^+^ (250 *μ*mol/L), isoflavone (10 *μ*M), isoflavone (10 *μ*M) + MPP^+^ (250 *μ*mol/L), Z-YVAD-CHO (10 nM) + MPP^+^ group, and Z-DEVD-CHO (10 nM) + MPP^+^ group. Cell viability was measured by MTT methods; the content of tyrosine hydroxylase was measured by immunocytochemistry method of avidinbiotin peroxidase complex; apoptosis ratio was measured by flow cytometry. The results showed that cell viability in the MPP^+^ group was lower than in all other five groups. There was no difference in cell viability between isoflavone + MPP^+^ and control group. Optical density of TH positive cells in isoflavone group was higher than in control, isoflavone + MPP^+^, and MPP^+^ only groups. The apoptosis ratio in the isoflavone + MPP^+^ group and control group and the Z-YVAD-CHO + MPP^+^ and Z-DEVD-CHO + MPP^+^ group was similar, which was lower than in the MPP^+^ group. The lowest apoptosis ratio was found in the isoflavone only group.

## 1. Introduction

Isoflavone is one of ingredients from soybean. Studies [1–3] have showed that isoflavone has some effect of estrogen and has many potential clinical implications with mechanism of action, especially in the treatment and prevention of diabetes, cardiovascular diseases, cancer, osteoporosis, and neuroprotection. Our previous study [4] indicated that estrogen had the protective effect to cell model of Parkinson's disease, but estrogen perhaps brings some side effects and restricts its use on clinical, so many researchers are looking for better substitute for estrogen, which not only has the effect of estrogen, but also has no side effect of it; fortunately researchers found plant-estrogen [1]. Isoflavone is one of the plant estrogens which has the same effect. And in this study, we use the cell model of Parkinson's disease to study whether isoflavone has the protective effect and explores the mechanism. More and more evidences indicate that apoptosis is the basic mechanism of neuron degeneration [5]. The active metabolic outcome of 1-methyl-4-phenylpyridinium (MPP^+^), MPTP could induce the apoptosis of PC12 cells [5, 6]. There are many studies indicating that [7, 8] MPP^+^ could induce apoptosis transmitted by caspase [9]. In this study, we observed the influence of isoflavone on cell model of Parkinsonism and how the caspase-1 and caspase-3 play a role in the course of injury induced by MPP^+^ in PC12 cells, in order to explore the protective mechanism of isoflavone to Parkinson's disease.

## 2. Materials and Methods

### 2.1. Cell Culture

PC12 cells were cultured at 37°C in RPMI 1640 media supplemented with 10% FCS, 2 mM of L-glutamine, 100 IU/mL of penicillin, and 100 *μ*g/mL of streptomycin in a humidified atmosphere of 5% carbon dioxide in room air. The dispersed cells were plated onto collagen-coated 96-well plates at a density of 3 × 10^4^ cells/well and were cultured under various combinations of times and drug regimens.

The cultured cells were divided into six groups: control (vehicle), MPP^+^ (250 *μ*mol/L) only, isoflavone (10 *μ*M) + MPP^+^ (250 *μ*mol/L) group, isoflavone (10 *μ*M) only group, Z-YVAD-CHO (10 mM) + MPP^+^ group, and Z-DEVD-CHO (10 mM) + MPP^+^ group. (Z-YVAD-CHO is the inhibitor of caspase-1; Z-DEVD-CHO is the inhibitor of caspase-3.) Using the method reported by Christis Chinopiulos and Vera Adam-Vizi, as we have reported in our previous work [6], the PC12 cells in the MPP^+^ group were treated with 250 *μ*mol/L of MPP^+^, inducing apoptosis similar to that seen in Parkinson's disease. In the isoflavone group, cells were cultured with isoflavone at a concentration of 10 *μ*M.

### 2.2. Thiazolyl Blue Tetrazolium Bromide (MTT) Assay of Cell Viability

After the PC12 cells were treated with MPP^+^ solution (5 mg/mL, Sigma) and different dose of isoflavone (0, 5 *μ*M, 10 *μ*M, 50 *μ*M). Incubation at 37°C for 4 h, formazan cuystal was dissolved in 100 *μ*l dimethyl sulfoxide (DMSO), and MTT reduction was measured at 570 nm using a DG-3022A ELISA plate reader. Control values were taken as 100%, and experimental values were taken as a percentage decrease in MTT reduction. The cell viability and metabolite were evaluated by A570.

### 2.3. Immunocytochemistry of Tyrosine Hydroxylase (TH)

This was performed to assess the levels of catecholamine biosynthesis in the PC12 cells. Sections were incubated with 0.3% Triton X-100 in PBS for 1 h at room temperature and then incubated with goat anti-rat TH monoclonal antibody (1 : 250 dilutions in 0.01 mmol/L phosphate buffer saline, PH 7.4), was added overnight at 4°C. Slides were then incubated with biotinylated rabbit anti-goat IgG and SABC-reagent for 30 min at 37°C. Subsequently, the cells were processed with 50 ul of 3,3′-diaminobenzidine (DAB) solution (Sigma). Cells were stained with hematoxylin and dehydrated with ethanol. Under a microscope, cells with dark brown color are considered as positive expression of tyrosine hydroxylase (TH), while light purple is considered as the negative.

### 2.4. Western Blot for Caspase-1 and Caspase-3

Cells were harvested by mechanical scraping into 4°C PBS solution, and add the cell lysis buffer into cells then centrifugate at 12000 g for 5 min. then acquired the upper as protein. Protein concentrations were determined by the BioRad protein assay. Thirty micrograms of protein was loaded per well onto 8% SDS-PAGE gel. Protein extracts were electrophoresed and transferred to a PVDF membrane. The membranes were blocked in 5% milk without fat and incubated in primary antibody for caspase-1 (1 : 2000, Sigma) and caspase-3 (1 : 2000, Sigma) and *β*-actin (1 : 2000, Sigma). The membranes were then washed in TBS plus 0.1% Tween-20 and incubated with HRP-conjugated secondary antibody, followed by another wash. The membranes were then developed with ECL reagent and exposed on film.

### 2.5. Detection of Apoptotic Cells by Flow Cytometry

The apoptosis rate of endothelial cells was measured by DNA flow cytometry and DNA electrophoresis (Table 2). Annexin V binding was assessed using bivariate flow cytometry, and cell staining was evaluated with fluorescein isothiocyanate- (FITC-) labelled Annexin V (green fluorescence), simultaneously with dye exclusion of propidium iodide (PI) (negative for red fluorescence). In each group, the total cells and the surviving cells were also counted in five and the mean values were derived.

### 2.6. Statistical Analysis

Data were expressed as means ± SD. Statistical analysis of the data for multiple comparison was performed by ANOVA. For single comparison, Student's *t*-test was used. Categorical data were analyzed with Chi-square test. *P* < 0.05 was considered statistically significant.

## 3. Results

### 3.1. Cell Viability

**Figure 1:**
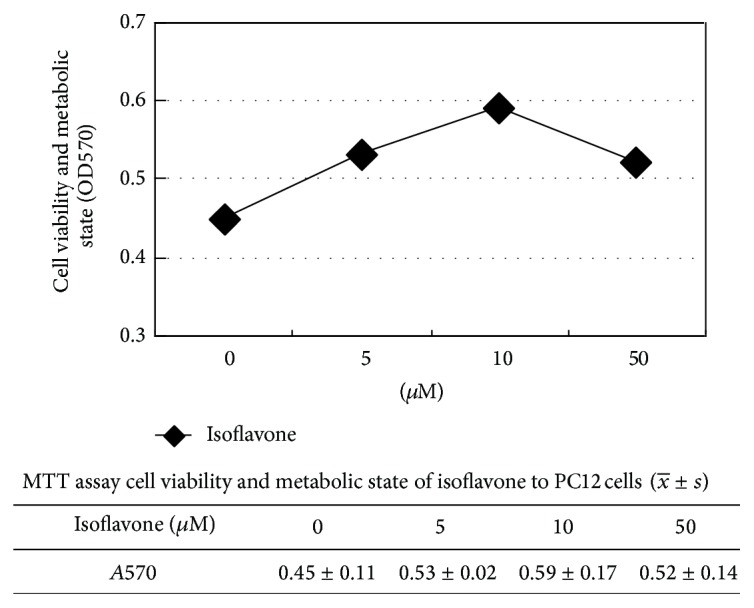


As shown in Figure 1, cell viability in I (10 *μ*M) group was higher than others, so we choose I (10 *μ*M) as the effective dose.

As shown in Table 1, cell viability in MPP^+^ group was lower than in control (*P* < 0.05), isoflavone + MPP^+^ (*P* < 0.01), Z-YVAD-CHO + MPP^+^, and Z-DEVD-CHO + MPP^+^ groups (*P* < 0.05). There was no significant difference in the cell viability between isoflavone + MPP^+^ and control group (*P* > 0.05).

### 3.2. Apoptosis and the Average Absorbency of TH Positive PC12 Cells

**Figure 2:**
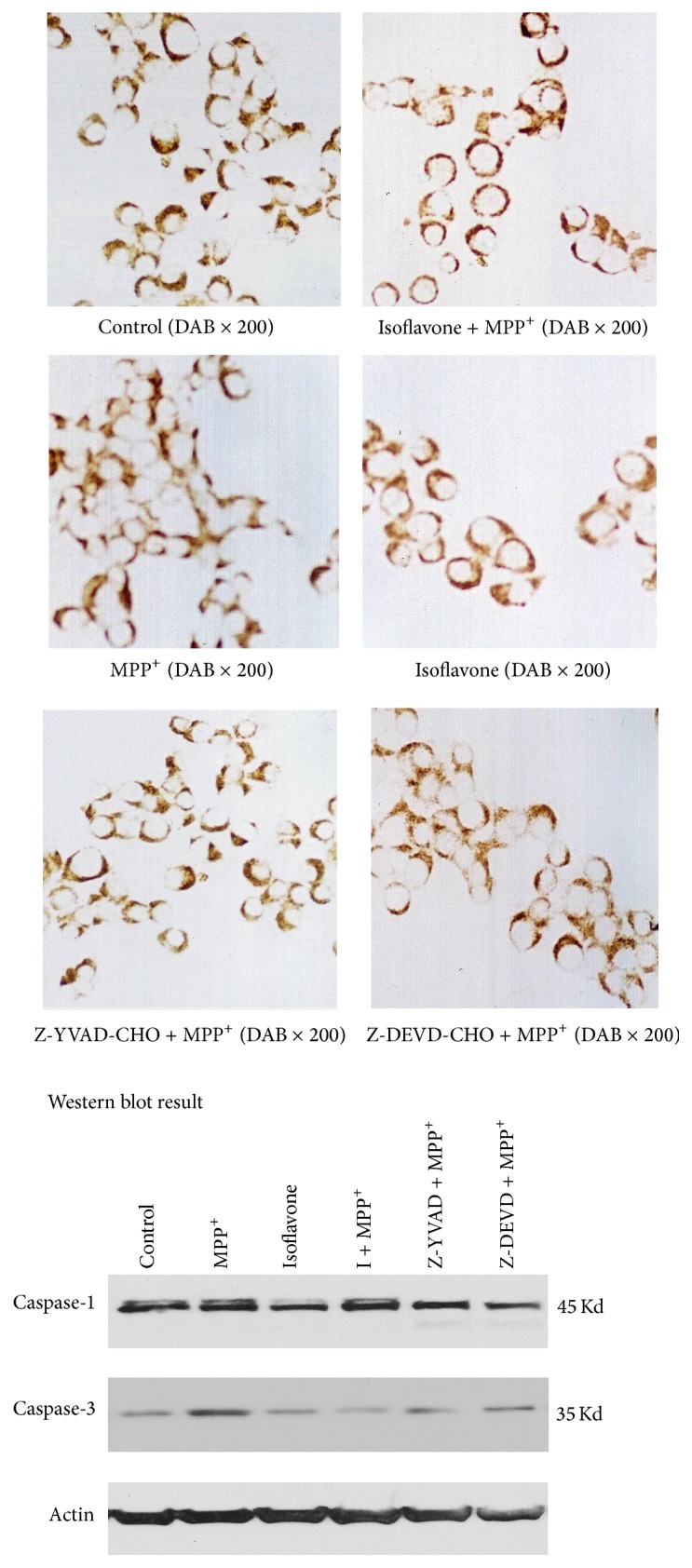


The TH positive cells were stained in brown color. The unstained nuclei in the TH positive cells were large and were rounded, with an empty appearance (Figure 2). The cells treated with isoflavone were larger than the cells in the control or MPP^+^ group, and most isoflavone-treated cells had neuritis (Figure 2). Some very small and round cells were detected in the MPP^+^ group (Figure 2).

Optical density in the TH positive cells in the isoflavone group was higher than in the control (0.46 ± 0.06 versus 0.22 ± 0.07, *P* < 0.05), the isoflavone + MPP^+^ (0.24 ± 0.04, *P* < 0.05), and the MPP^+^ only group (0.10 ± 0.03, *P* < 0.05). There was no significant difference between the control and isoflavone + MPP^+^ group (*P* < 0.05).

The apoptosis ratio in isoflavone + MPP^+^ (33.6%) group and control group (31.3%) and the Z-YVAD-CHO + MPP^+^ (34.2%) and Z-DEVD-CHO + MPP^+^ group (35.6%) was similar (*P* > 0.05, Table 1), which was lower than in the MPP^+^ group (63.5%) (*P* < 0.05, Table 1). The lowest apoptosis ratio was found in the isoflavone only group (11.5%, *P* < 0.05, Table 1).

### 3.3. Level of Caspase-1 and Caspase-3 Protein

The levels of protein of caspase-1 and caspase-3 are higher in MPP^+^ group than in control (*P* < 0.05), isoflavone + MPP^+^ (*P* < 0.01), Z-YVAD-CHO + MPP^+^, and Z-DEVD-CHO + MPP^+^ groups (*P* < 0.05). There was no significant difference between isoflavone + MPP^+^ and control group (*P* > 0.05).

## 4. Discussion

The loss of dopamine-producing nerve cells in the substantia of the midbrain is the main pathological characteristics of PD [10]. A study found that the loss of midbrain neurons was positively related to caspase-3 positive neurons when using immunohistochemistry method for autopsy in PD patients [11]. The levels of caspase-1 and caspase-3 were elevated in the dopaminergic neurons of the substantia compact part which were degenerative [12]. In a mouse model of subchronic PD made by MPTP, the activation of caspase-3 reached the peak in the first two days, but the loss of dopaminergic neurons was not obvious until the seventh day [12].

The previous study suggested that caspase activation was the early stage signal of dopaminergic neurons in the process of apoptosis. In this study, we add MPP^+^ to PC12 cells and induced cell apoptosis model similar to neuron damage in PD. We have observed the influence of the isoflavone and caspase-1 and caspase-3 inhibitors on PC12 cell apoptosis. There is no statistically significant difference in cell viability, apoptosis rate, and TH optical density between the isoflavone + MPP^+^ (*P* < 0.01), Z-YVAD-CHO + MPP^+^, and Z-DEVD-CHO + MPP^+^ groups (*P* < 0.05) and control group cells, but the MPP^+^ group is lower than all the other groups, and the isoflavone group is higher than all the other groups. Caspase-1 and caspase-3 protein level is higher in MPP^+^ group than all the other groups, and the isoflavone group is lower than all the other groups. There is no significant difference between MPP^+^ + isoflavone group and caspase inhibitors + MPP^+^ group and MPP^+^ group on the cell activity, TH optical density, apoptosis rate, and caspase-1 and caspase-3 protein level. Caspase inhibitors group's cell activity and TH optical density are significantly elevated, but the apoptosis rate is significantly reduced. This result coincides with the previous study [13]. The interleukin-1 beta protease family in the Mammals and ced-3 gene production which control nematodes apoptosis are highly conserved. The family protein has cysteine protein enzymes and aspartate specific enzymes, also named cysteine aspirate specific protease caspase. Numerous studies indicate that caspase mediates neuron's apoptosis in the neural degenerative disease, but the enzyme can save neurons's death process in the apoptosis stimulation process. Caspase-1 and caspase-3 are the two of 14 caspase family members, which have close relationship with the apoptosis [14]. Caspase-3 is the ced-3 related cysteine protease, which is heterodimer, made by the 28 kD proenzyme's zymolysis, composing 17 and 12 kd subunits. It is the key enzyme in the early stage activation and directly mediates the effect of apoptosis in the downstream. Caspase-3 is called “apoptosis executives” as the most direct gene on regulating apoptosis. MPP^+^ can activate apoptosis cascade reaction, reduce the mitochondrial membrane potential, accelerate tumor necrosis factor (TNF) transcript, and activate caspase-3 to open holes in the mitochondrial membrane, enhancing its permeability, which eventually lead to dopaminergic neuron's apoptosis. So we tentatively put forward that isoflavone and caspase inhibitors can prevent cell death and Isoflavone may play the role similar to caspase inhibitors in protecting and directly suppresses “apoptosis executives” activities, which effectively resist apoptosis's occurring [15]. So the result may be due to the fact that isoflavone suppressed MPP^+^-induced apoptosis in PC12 cells. The apoptosis suppression is associated with suppressing caspase-1 and caspase-3.

Recent research indicates that [8, 16] caspase inhibitors can not only restrain cell death, but also save axonal loss and the reduction of 3H take-in. Caspase inhibitors may make neurons immortal, but it is not effective for its functional recovery. But because the synthetic caspase inhibitors cannot go through blood brain barrier, so limit its clinic application. But numerous studies have shown that isoflavone can protect nerve by changing cell survival, axon extension, and enhancing neurotransmitter transmitting, which reflects obvious superiority [12, 13]. The wide application and safety of plant-based estrogens provide good prospect for estrogen in the clinical treatment of PD. We have to point out that there is a long way to study the effect and mechanism further.

## Figures and Tables

**Table 1:** MTT assay of cell viability and optical density of TH positive PC12 cells.

Group	Cell viability	Optical density of TH positive PC12 cells
Control	0.49 ± 0.11	0.22 ± 0.07
MPP^+^	0.30 ± 0.07^**^	0.10 ± 0.03^**^
I (isoflavone)	0.61 ± 0.17^*^	0.46 ± 0.06^*^
I + MPP^+^	0.56 ± 0.16	0.24 ± 0.04
Z-YVAD-CHO + MPP^+^	0.59 ± 0.17^***^	0.22 ± 0.05^***^

Z-DEVD-CHO + MPP^+^	0.60 ± 0.11^***^	0.23 ± 0.02^***^

^*^ *P* < 0.05 compared with isoflavone + MPP^+^; ^**^ *P* < 0.05 compared with control; ^***^ *P* < 0.05 compared with MPP^+^.

**Table 2:** Apoptosis rate (%) of PC12 cells.

Group	Apoptosis rate (%)	Necrosis rate (%)	Alive rate (%)

Control	31.3 ± 3.6	3.6 ± 0.3	65.1 ± 4.3
MPP^+^	63.5 ± 3.1^**^	4.3 ± 0.4	32.2 ± 3.4
Isoflavone (I)	11.5 ± 2.8^*^	0.8 ± 0.2	87.7 ± 3.8
I + MPP^+^	33.6 ± 3.7	5.1 ± 1.6	61.3 ± 5.6
Z-YVAD-CHO + MPP^+^	34.2 ± 1.8^***^	3.8 ± 0.7	61.0 ± 4.1

Z-DEVD-CHO + MPP^+^	35.6 ± 2.5^***^	4.0 ± 0.2	60.4 ± 3.7

^*^ *P* < 0.05 compared with isoflavone + MPP^+^; ^**^ *P* < 0.05 compared with Control; ^***^ *P* < 0.05 compared with MPP^+^.
